# Magnetic Field/Ultrasound-Responsive Fe_3_O_4_ Microbubbles for Targeted Mechanical/Catalytic Removal of Bacterial Biofilms

**DOI:** 10.3390/nano14221830

**Published:** 2024-11-15

**Authors:** Liang Lu, Yuan Liu, Xiaolong Chen, Fengjiao Xu, Qi Zhang, Zhaowei Yin, Lihui Yuwen

**Affiliations:** 1 State Key Laboratory of Organic Electronics and Information Displays, Jiangsu Key Laboratory of Smart Biomaterials and Theranostic Technology, Institute of Advanced Materials (IAM), Nanjing University of Posts and Telecommunications, Nanjing 210023, China; 2 Department of Orthopaedic, Nanjing First Hospital, Nanjing Medical University, Nanjing 210006, China

**Keywords:** bacterial biofilm infection, ultrasound, microbubbles

## Abstract

Conventional antibiotics are limited by drug resistance, poor penetration, and inadequate targeting in the treatment of bacterial biofilm-associated infections. Microbubble-based ultrasound (US)-responsive drug delivery systems can disrupt biofilm structures and enhance antibiotic penetration through cavitation effects. However, currently developed US-responsive microbubbles still depend on antibiotics and lack targeting capability. In this work, magnetic field/ultrasound (MF/US)-responsive Fe_3_O_4_ microbubbles (FMB) were constructed based on Fe_3_O_4_ nanoparticles (NPs) with superparamagnetic and peroxidase-like catalytic properties. In vitro experiments demonstrated that FMB can be targeted to methicillin-resistant Staphylococcus aureus (MRSA) biofilms by the direction of MF. Upon US irradiation, FMB collapse due to inertial cavitation and generate mechanical forces to disrupt the structure of MRSA biofilms and releases Fe_3_O_4_ NPs, which catalyze the generation of reactive oxygen species (ROS) from H_2_O_2_ in the biofilm microenvironment and kill the bacteria within the biofilm. In a mouse biofilm infection model, FMB efficiently destroyed MRSA biofilms grown in subcutaneous catheters with the MF and US. Magnetic-targeted mechanical/catalytic therapy based on FMB provides a promising strategy for effectively combating bacterial biofilm infection.

## 1. Introduction

Bacterial biofilms are densely arranged bacterial communities where bacteria encapsulate themselves by forming extracellular polymeric substances (EPS) [1,2]. Biofilms can form on the surface of human tissues and implanted devices, leading to persistent infectious diseases [3]. Clinically, more than 80% of chronic bacterial infections are associated with biofilm formation [4,5]. The physicochemical barriers of EPS provide multiple protective functions. They enhance the mechanical strength of the biofilm, impede the penetration of antibiotics, and lead to high drug resistance, which poses a great challenge to the traditional antibiotic treatment of biofilm infections [6]. Currently, clinical treatments of bacterial biofilm infections mainly involve surgical removal and the use of high doses of antibiotics [7,8,9]. However, these methods have limited effectiveness and serious side effects. Therefore, there is an urgent need for alternative non-antibiotic methods to destroy biofilms and kill the bacteria within them.

Recently, US-responsive drug delivery systems with mechanical disruption properties have demonstrated their potential for eliminating bacterial biofilms [10,11,12,13,14]. US not only offers advantages, such as tissue penetration, biosafety, and controllability, but can also enhance the penetration of antibacterial agents through the cavitation effect-induced biofilm disruption [15]. He et al. and Dong et al. demonstrated that US-responsive microbubbles can facilitate the penetration of vancomycin into Staphylococcus epidermidis biofilms and improve the antibacterial effect [16,17]. Huang et al. loaded meropenem onto polymer-modified perfluoropentane nanodroplets, which effectively enhanced drug penetration under US exposure and improved the clearance of Pseudomonas aeruginosa biofilms [18]. Our group fabricated US-responsive Fe_3_O_4_-piperacillin composite microbubbles for biofilm eradication. Under US stimulation, these drug-loaded microbubbles disrupted the Pseudomonas aeruginosa biofilm structure through mechanical force and enhanced the penetration of piperacillin within the biofilm, effectively treating chronic pulmonary infections in mice [19]. Nevertheless, US-responsive antibiofilm agents still suffer from the lack of biofilm targeting and reliance on antibiotics. Therefore, there is an urgent need to develop novel antibiofilm agents and strategies that can target and destroy the biofilm without inducing drug resistance.

Nanocatalytic therapy (NCT) is an emerging therapeutic modality that employs nanocatalysts to generate ROS for pathogen elimination [20,21]. ROS can kill bacteria by oxidizing their vital biological molecules and can prevent the development of bacterial drug resistance [22]. For instance, Fe_3_O_4_ NPs possess peroxidase-like activity and can catalyze the generation of hydroxyl radicals (•OH) from H_2_O_2_ under slightly acidic conditions, leading to bacterial death [10,23]. Gao et al. reported that Fe_3_O_4_ NPs with peroxidase-like activity degraded the main components of bacterial biofilms [24]. Fe_3_O_4_ NPs exhibited an antibacterial rate of over 99.999% (5 Log) against Streptococcus mutans biofilms in the presence of H_2_O_2_. Dong et al. prepared porous Fe_3_O_4_ nanoparticles (p-Fe_3_O_4_), which achieved an antibacterial rate of over 99.99% against Escherichia coli and Bacillus cereus biofilms with 100 mM H_2_O_2_ [23]. Du et al. prepared Fe_3_O_4_ NPs-glucose oxidase nanomaterials that can initiate a cascade catalytic reaction in the acidic biofilm microenvironment (pH~5.5) and generate •OH for biofilm removal. Despite the promising potential of NCT in the clearance of bacterial biofilms, current nanocatalysts still suffer from issues, such as the lack of targeting and insufficient permeability, which limit the efficiency of NCT in biofilm eradication. Therefore, the development of US-responsive antibiofilm agents that combine biofilm targeting, mechanical disruption, and catalytic antibacterial capabilities is expected to address these problems.

**Figure 1:**
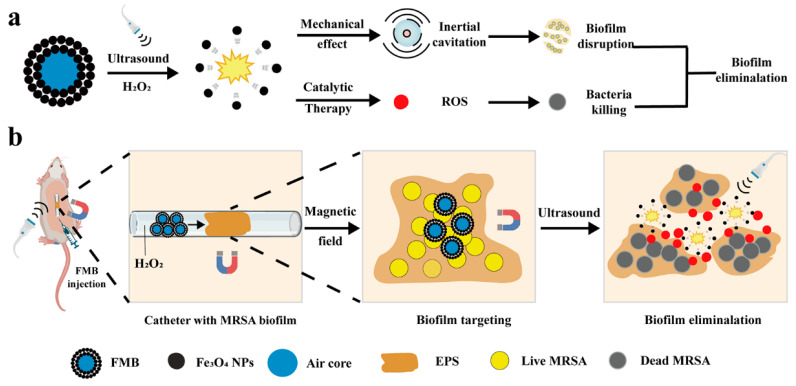
Schematic diagram of magnetic field/ultrasound (MF/US)-responsive Fe_3_O_4_ microbubbles (FMB) for bacterial biofilm removal. (**a**) Mechanism of action of FMB for MF-targeted mechanical/catalytic removal of bacterial biofilms. (**b**) FMB target the MRSA biofilm of mouse subcutaneous catheter under the guidance of MF, destroy the biofilm structure by ultrasound cavitation effect, and catalyze the production of ROS from H_2_O_2_ to kill the bacteria in the biofilm.

In this work, we assemble Fe_3_O_4_ NPs with superparamagnetic and peroxidase-like catalytic properties to construct US-responsive FMB for MRSA biofilm elimination. Under the guidance of MF, FMB target and accumulate at the biofilm site. Upon US irradiation, FMB undergo inertial cavitation, generate mechanical force, disrupt the biofilm structure, and enhance the penetration of Fe_3_O_4_ NPs into biofilms (Figure 1a). The released Fe_3_O_4_ NPs can catalyze H_2_O_2_ to generate ROS for killing bacteria. As depicted in Figure 1b, FMB utilizes both the acoustic effects of microbubble and the catalytic effects of Fe_3_O_4_ NPs for MF-targeted biofilm elimination. The biofilm elimination performance of FMB was evaluated using both the in vitro and in vivo biofilm models.

## 2. Materials and Methods

### 2.1. Materials and Reagents

Anhydrous glucose, sodium chloride (NaCl), Triton X-100 (Triton), methylene blue (MB), and hydrogen peroxide (H_2_O_2_, 10 M) were purchased from Sinopharm, Shanghai, China. Sodium dodecyl sulfate (SDS) and 3,3′,5,5′-tetramethylbenzidine (TMB) were supplied by Sigma-Aldrich, St. Louis, MO, USA. Luria–Bertani (LB) medium, LB agarose medium, and CCK-8 cell proliferation and cytotoxicity kits were purchased from Beyotime Biotechnology, Haimen, China. Iron oxide nanoparticles (Fe_3_O_4_ NPs, 50–100 nm) were purchased from Alfa Aesar, Ward Hill, MA, USA and were used without surface functionalization. Crystal violet staining solution (2%), Calcein acetoxymethyl ester (Calcein–AM), DMEM medium, and PBS buffer were provided by Keygen Biotech, Nanjing, China. Fetal bovine serum (FBS) was purchased from Gibco, Grand Island, NY, USA. Universal tissue fixative was purchased from Wuhan Google Biotechnology, Wuhan, China. Ultrapure water was used in all experiments of this study.

### 2.2. Preparation of FMB

In a centrifuge tube, 30.0 mg of Fe_3_O_4_ NPs and 400 μL of ultrapure water were added sequentially and mixed for 30 s using a vortex mixer. Then, 150 μL of SDS aqueous solution (10.0 mM), and 150 μL of ultrapure water were added sequentially. The centrifuge tubes were placed in an ice-water bath, and the bubbles were formed by high-speed stirring (10,000 rpm, 3 min) through a homogenizer (D-160, DLAB). The FMB were allowed to stand at 4 °C for 8 h and were finally magnetically separated. The resulting FMB was washed three times using ultrapure water.

### 2.3. MF/US-Responsive Properties of FMB

MF-responsive properties: FMB (Fe_3_O_4_ NPs: 1.0 mg/mL) was dispersed in a glass vial containing 2 mL of ultrapure water, and the movement of FMB was photographed under MF using a magnet (8 mT) close to the bottom of the vial.

MF-targeting properties: FMB (Fe_3_O_4_ NPs: 0.5 mg) was added to a medical silicone catheter filled with ultrapure water, and after bending the silicone catheter into a complex structure, a permanent magnet (8 mT) was used to attract FMB through the bend to reach the targeting position.

US-responsive properties: FMB (Fe_3_O_4_ NPs: 1.0 mg/mL) were dispersed in a glass vial containing 2 mL of ultrapure water and were irradiated under the US for 5 min at 1.0 W/cm^2^, 1 MHz, and 50% duty cycle, and the morphology change of FMB was photographed.

### 2.4. Catalytic Effects of FMB

TMB was used as a chemical probe to verify the catalytic effect of FMB. TMB solution (10 mg/mL), NaAc–HAc buffer (pH 4.0), H_2_O_2_ solution (10.0 M), and FMB aqueous dispersion (Fe_3_O_4_ NPs: 1.0 mg/mL) were used. For the TMB + H_2_O_2_ + FMB + US group, FMB dispersion, NaAc–HAc buffer, H_2_O_2_ solution, and TMB solution were added to centrifuge tubes and irradiated by US (1.5 W/cm^2^, 1 MHz, 50% duty cycle, 5 min). The centrifuge tubes were shaken (600 rpm, 5 min) at 37 °C. After the reaction was completed, the supernatant was taken after magnetic separation. The ultraviolet-visible absorption spectra of the supernatant were measured.

### 2.5. MF-Targeted Mechanical Disruption of Biofilms

The destructive properties of FMB on MRSA biofilms were investigated under different conditions. The 96-well plates with MRSA biofilms were grouped as follows: Saline group, 100 µL saline; FMB group, 50 µL saline + 50 µL FMB (Fe_3_O_4_ NPs: 1.0 mg/mL). The ultrasound probe (5 cm^2^) covered with medical ultrasound coupling agents was placed to the bottom of 96-well plates with MRSA biofilms to apply the ultrasound (1.0 W/cm^2^, 1 MHz, 50% duty cycle). At the same time, the ring magnet was placed around the ultrasound probe to provide a magnetic field (32 mT). After treatment, saline (100 μL) was added for rinsing. The residual Fe_3_O_4_ NPs on the surface of the biofilm were removed by using a magnetic bar, and the washing was repeated three times. Afterwards, the solution in the wells was removed, and 4% paraformaldehyde solution was added for fixation. Then, the supernatant was removed and the plates were allowed to dry naturally at room temperature. Crystal violet staining solution was added to each well to stain the biofilm. Then, the staining solution was removed and the plate was rinsed by a slow stream of water. The MRSA biofilm was imaged by an inverted fluorescence microscope.

For the relative biofilm biomass assay of MRSA biofilms, ethanol (95%) was added to each well to decolorize the crystal violet-stained MRSA biofilm and the absorbance of the solution at 590 nm was measured.

### 2.6. Mechanical/Catalytic Destruction of MRSA Biofilms

MF-targeted catalytic disruption of MRSA biofilms by FMB with the presence of MF, US, and H_2_O_2_. MRSA biofilms in 96-well plates were grouped according to the concentration of H_2_O. The procedures and parameters used in this experiment were the same as those used in Section 2.5, except for the addition of H_2_O_2_.

### 2.7. Antibacterial Effect on MRSA Biofilms

To study the antibacterial effect of FMB on MRSA biofilms, the 96-well plates with MRSA biofilms were divided into Control, MF + US, FMB, H_2_O_2_, FMB + H_2_O_2_, and FMB + H_2_O_2_ + MF + US groups. The concentration of FMB saline dispersion and H_2_O_2_ solution was 1 mg/mL (Fe_3_O_4_ NPs) and 600 mM, respectively. After the FMB was added to the well, MF was exerted by a magnet (32 mT), and US (1.0 W/cm^2^, 1 MHz, 50% duty cycle) was used for a total action time of 10 min. Subsequently, the 96-well plate was incubated at 37 °C for 6 h. After incubation, the liquid in the well was removed and added to a sterilized centrifuge tube. Then, saline was added to repeatedly rinse the biofilm, and the dispersion was transferred to the centrifuge tube. Subsequently, the well was sonicated in a water bath, and the biofilm dispersion after sonication was transferred to the centrifuge tube. The above steps were repeated until the dispersion was clear. The biofilm dispersion was sonicated in a water bath, vortexed, and diluted. The number of viable bacteria in the MRSA biofilm was determined by the plate counting method.

### 2.8. Fluorescence Imaging of MRSA Biofilms

MRSA biofilms were grown in laser confocal dishes and divided into six groups according to the treatment conditions: the Control group and the US + MF group were added with PBS; the FMB group was added with PBS and FMB dispersion; the H_2_O_2_ + FMB group was added with PBS, FMB dispersion, and H_2_O_2_; and the H_2_O_2_ + FMB + US + MF group was added with PBS and FMB. After treatment, the confocal dish was incubated at 37 °C for 30 min. Calcein–AM solution (4 μM) was added and incubated for 30 min at 37 °C. The fluorescence image was observed and photographed on a confocal laser scanning microscope (CLSM).

### 2.9. Culture of MRSA Biofilms on Catheters

Sterilized medical silicone catheters were incubated with FBS solution (1%). After the FBS solution was removed, MRSA bacterial suspension (10^7^ CFU/mL) was added into the catheter and incubated at 37 °C for 48 h to obtain the MRSA biofilm.

### 2.10. Removal of MRSA Biofilms from Catheters In Vitro

The medium was removed from the silicone catheter with biofilms and sterilized saline was added. The magnet was placed near the catheter. After the addition of FMB dispersion, the magnet was moved to the inner part of the catheter with biofilms. The ultrasound (1 MHz, 50% duty cycle) was performed at 1.0 W/cm^2^ for 10 min. For crystal violet staining, the liquid in the catheter was removed, and 4% paraformaldehyde was added. After fixation, the fixative was removed, and the catheter was left to dry. Then, the crystal violet staining solution was added to the catheter. After 30 min, the staining solution was removed. The catheter was repeatedly rinsed with saline. The crystal violet in the biofilm was dissolved by ethanol. The absorbance of the decolorized solution at 590 nm was measured.

For the antibacterial effect study, the liquid inside the catheter after treatment was transferred to a sterilized centrifuge tube. Subsequently, saline was added to the catheter, sonicated for 3 min, and transferred to the centrifuge tube. The washing procedure was repeated several times until all the biofilms were collected. Finally, the biofilm dispersions were diluted, sonicated, and vortexed. The number of viable bacteria in the MRSA biofilm was investigated by plate counting method.

### 2.11. Removal of MRSA Biofilms from Catheters In Vivo

All animal experiments were performed following the guidelines for the care and use of laboratory animals from the Nanjing First Hospital, Nanjing Medical University, and approved by the Animal Ethics Committee of Nanjing First Hospital, Nanjing Medical University. A mouse catheter biofilm model was established subcutaneously in BALB/c mice (female, 6–8 weeks). Catheters with MRSA biofilms were washed with saline and implanted under the skin of mice. Sixteen mice with catheters were randomly divided into two groups: the Control group and the FMB + US + MF group, with eight mice in each group.

For the FMB + US + MF group, the magnet was placed close to one end of the catheter. FMB and H_2_O_2_ were injected into the other end of the catheter by using a syringe. Then, US (1.0 W/cm^2^, 1 MHz, 50% duty cycle) was performed with a 5 min interval. For the Control group, catheters were rinsed twice using saline. After treatment, the mice were anesthetized and executed. The catheters were removed and rinsed using saline. Catheters from four mice were used for crystal violet staining study, and catheters from the other four mice were used for the antibacterial effect study.

For the crystal violet staining study, the liquid in the catheters was removed, and 4% paraformaldehyde solution was added. After the fixative was removed, the catheter was dried naturally. Afterward, crystal violet staining solution was added for 30 min. After the staining solution was removed, the catheter was rinsed by adding saline, and the MRSA biofilm was photographed with a camera. Subsequently, the crystal violet in the biofilm was decolorized with ethanol, and the absorbance of the decolorized solution at 590 nm was measured to calculate the relative biofilm biomass.

To investigate the antibacterial effect, the catheter was washed and sonicated repeatedly to disperse the biofilm in saline. Then, the biofilm dispersions were transferred to a centrifuge tube, sonicated for 5 min, and vortexed for 20 s. Finally, the number of viable bacteria was determined by the plate counting method.

### 2.12. Statistical Analysis

All data are presented as means ± standard deviations (S.D.). One-way analysis of variance (ANOVA) with Tukey’s post hoc test was used for statistical analysis (* *p* < 0.05, ** *p* < 0.01, and *** *p* < 0.001).

## 3. Results

### 3.1. Characterization of FMB

**Figure 2:**
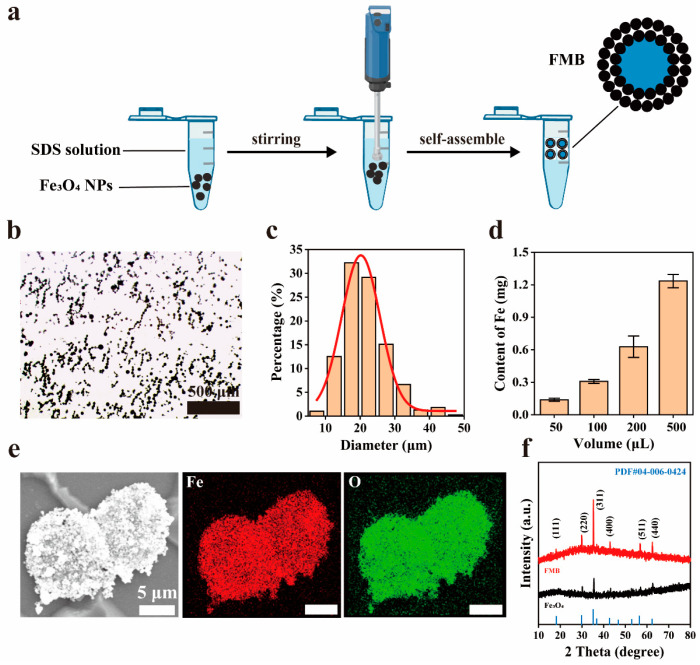
Preparation and characterization of FMB. (**a**) Schematic of the preparation process of FMB. (**b**) Bright-field microphotograph of FMB. (**c**) Size distribution histogram of FMB with a statistical number greater than 200. (**d**) Content of Fe in different volumes of FMB aqueous dispersion. (**e**) Scanning electron microscopy (SEM) images and elemental mapping images of FMB. (**f**) X-ray diffraction (XRD) spectra of Fe_3_O_4_ NPs, FMB, and standard powder diffraction pattern of Fe_3_O_4_ (PDF#04-006-0424).

As shown in Figure 2a, hydrophobic Fe_3_O_4_ NPs self-assemble to form FMB at the air-water interface, assisted by SDS under high-speed rotary shear in the homogenizer. FMB exhibited a uniformly distributed spherical morphology with an average diameter of 23.5 ± 7.2 μm (Figure 2b,c). The Fe content in varying volumes of FMB aqueous dispersions increased proportionally with the volume. Each 50 μL of FMB aqueous dispersion contained approximately 0.14 mg Fe (Figure 2d).

SEM elemental mapping images showed that Fe and O elements were uniformly distributed in the FMB, indicating the successful assembly of Fe_3_O_4_ NPs (Figure 2e). As shown in Figure 2f, the XRD patterns of FMB exhibited strong diffraction peaks at 29.8°, 35.1°, and 56.4°, corresponding to the (220), (311), and (511) crystal planes of Fe_3_O_4_ (PDF#04-006-0424), respectively, indicating that there was no obvious change in Fe_3_O_4_ before and after the assembly.

### 3.2. US and MF Response Performance of FMB

**Figure 3:**
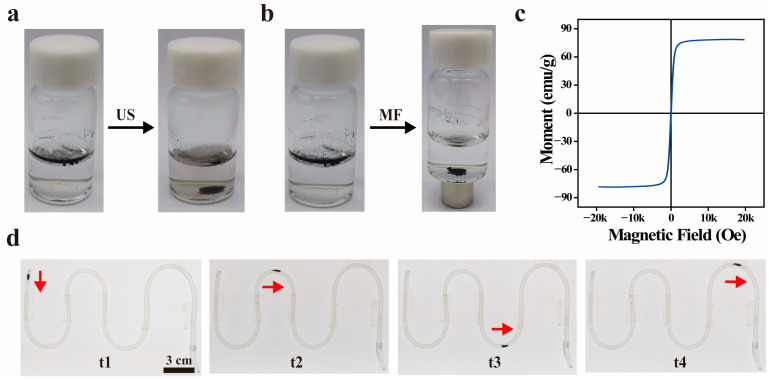
MF/US-responsive properties of FMB. (**a**) Photographs of FMB before and after ultrasound irradiation. (**b**) Photographs of FMB before and after the action of MF. (**c**) Vibrating sample magnetometer (VSM) spectra of FMB. (**d**) Photographs of FMB migrating to the target position in the catheter under the action of a permanent magnet. t1, t2, t3, and t4 represent different time points. The red arrow indicates the moving direction of FMB.

As shown in Figure 3a, before the application of US, the FMB had a spherical structure with a shell of Fe_3_O_4_ NPs wrapped around an air core, allowing them to float on the water surface. After US irradiation (1.0 W/cm^2^, 1 MHz, 50% duty cycle, 5 min), the FMB underwent inertial cavitation, causing the shell layer to rupture and release the Fe_3_O_4_ NPs before settling at the bottom of the glass vial. Before applying the MF, the FMB floated due to their microbubble structure. After applying the MF, the FMB overcame the upward buoyancy force and rapidly gathered near the permanent magnet at the bottom of the glass vial (Figure 3b). The magnetic saturation of the FMB was 78.7 emu/g, indicating that they have superparamagnetic properties (Figure 3c). As shown in Figure 3d, the FMB were added into a catheter filled with water. Under the influence of the MF, the FMB changed from a dispersed state to an aggregated state, advanced along the preset path, and finally arrived at the target location. Thus, the FMB has excellent US/MF-responsive properties.

### 3.3. Catalytic Properties of FMB

**Figure 4:**
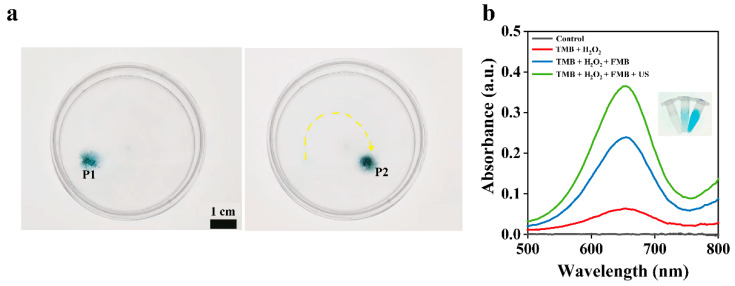
Catalytic properties of FMB. (**a**) Photographs of FMB catalyzing TMB in a Petri dish containing 300 mM H_2_O_2_. P1 and P2 represent the locations of FMB at different time points. The yellow arrow indicates the moving path of FMB. (**b**) Ultraviolet-visible-near-infrared (UV-vis-NIR) absorption spectra of FMB dispersions after reaction with TMB under different conditions; insets from left to right are photographs of TMB + H_2_O_2_, TMB + H_2_O_2_ + FMB, and TMB + H_2_O_2_ + FMB + US groups.

Fe_3_O_4_ NPs have peroxidase-like properties and can catalyze the generation of •OH from H_2_O_2_. The •OH can oxidize TMB to produce blue TMB oxides. As shown in Figure 4a, the blue product in the Petri dish containing PBS buffer indicates that the FMB catalyzed the generation of •OH during their movement from the P1 to P2 position. The yellow curve represents the trajectory of the FMB under the direction of the permanent magnet. The FMB after ultrasonication showed higher catalytic activity than those without ultrasonication (Figure 4b), which can be attributed to the disruption of the FMB structure and release of Fe_3_O_4_ NPs from the microbubbles, providing more catalytic sites.

### 3.4. Destruction of MRSA Biofilms by FMB in 96-Well Plates

**Figure 5:**
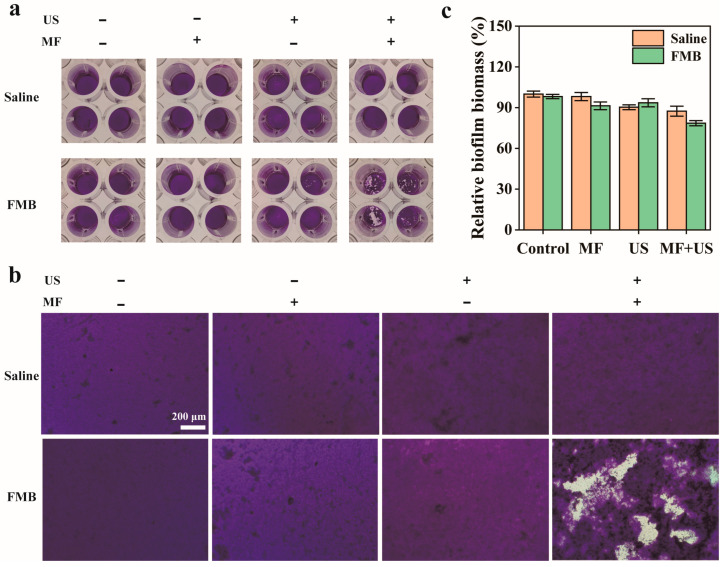
MRSA biofilm disruption by FMB under MF and US. (**a**) Optical photographs, (**b**) microphotographs, and (**c**) relative biofilm biomass of MRSA biofilms after crystal violet staining with different treatments.

We examined the destructive effect of Fe_3_O_4_ NPs on the MRSA biofilm structure under MF and US. As shown in Figure S1, Fe_3_O_4_ NPs could not effectively destruct MRSA biofilms under US and MF. We further examined the destructive effect of FMB on the MRSA biofilm structure under the influence of MF and US. As shown in Figure 5a,b, no significant damage to the biofilm structure was observed when MF, US, or MF + US treatments were applied without the addition of FMB. Similarly, in the FMB + MF group, no significant damage to the biofilm structure was observed. When US treatment was used, the biofilm structure was partially disrupted, indicating that mechanical forces generated by FMB can damage the biofilm structure. When both MF and US were simultaneously applied, the MF guided the FMB to bind to the biofilm surface, and the mechanical force generated under US could act directly on the nearby biofilm, thus, disrupting the biofilm more effectively. The relative biofilm biomass of the FMB + MF + US group was reduced the most (21.5%), which further demonstrates that FMB combined with MF/US can effectively destroy the biofilm structure (Figure 5c).

**Figure 6:**
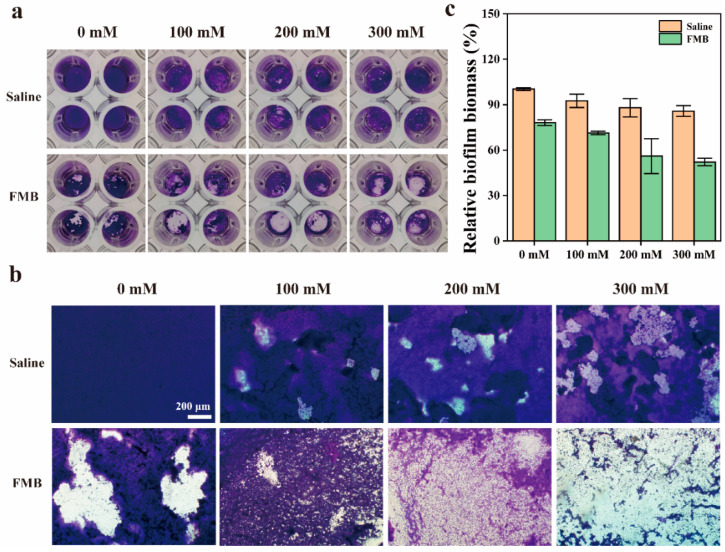
MRSA biofilm disruption by FMB under MF and US at different concentrations of H_2_O_2_. (**a**) Optical photographs, (**b**) microphotographs, and (**c**) relative biofilm biomass of MRSA biofilms grown in 96-well plates with crystal violet staining after different treatments.

Fe_3_O_4_ NPs have peroxidase-like activity and can catalyze the production of •OH from H_2_O_2_ in acidic microenvironments. As shown in Figure 6a,b, without the addition of FMB, there was no significant damage to the biofilm structure after treatment, even with increasing the H_2_O_2_ concentration. However, in the presence of FMB, damage to the biofilm structure became more significant as the H_2_O_2_ concentration increased. As shown in Figure 6c, when the H_2_O_2_ concentration increased from 0 mM to 300 mM, the relative biomass reduction of MRSA biofilms increased from 21.9% to 47.9% under FMB + MF + US treatment. These results demonstrate that MF/US-responsive FMB can effectively catalyze the production of •OH from H_2_O_2_ to degrade MRSA biofilms.

**Figure 7:**
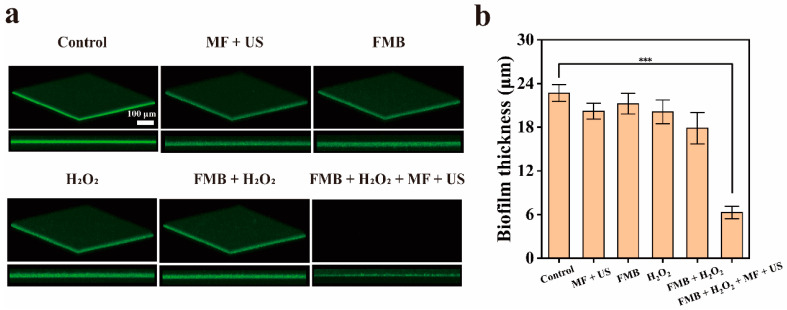
Fluorescence imaging of MRSA biofilms. (**a**) Three-dimensional confocal laser scanning microscopy (3D CLSM) photographs of MRSA biofilms stained by Calcein–AM after different treatments. (**b**) Thickness of MRSA biofilms after different treatments. *** *p* < 0.001.

As shown in Figure 7a,b, the biofilm thickness in the Control and MF + US groups did not change significantly, indicating that US or MF alone does not destroy the MRSA biofilms. Among the FMB, H_2_O_2_, and FMB + H_2_O_2_ groups, the smallest biofilm thickness of approximately 18 μm was observed in the FMB + H_2_O_2_ group, indicating that FMB combined with H_2_O_2_ can destroy the biofilm. In the FMB + H_2_O_2_ + MF + US group, the biofilm was extensively destroyed, and the biofilm thickness was reduced to about 7 μm, suggesting that FMB can efficiently remove MRSA biofilms through MF-targeted mechanical/catalytic effects with the combined action of the MF/US.

**Figure 8:**
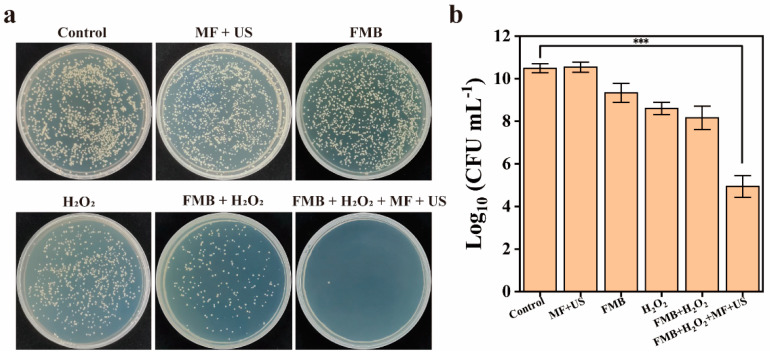
Antibacterial effect of FMB against MRSA biofilms. (**a**) Photographs of MRSA colonies on agar plates and (**b**) the number of viable bacteria in MRSA biofilms after different treatments. *** *p* < 0.001.

The antibacterial effect of FMB with or without MF/US on MRSA biofilms was further investigated. As shown in Figure 8a,b, in the US + MF group, there was no significant change in the number of bacterial colonies, indicating the neglectable antibacterial effect of US and MF. In the FMB group and H_2_O_2_ group, the number of MRSA was reduced by 1.0 Log (90.7%) and 1.2 Log (93.7%), respectively, indicating low antibacterial efficiency of FMB or H_2_O_2_ alone. In the FMB + H_2_O_2_ group, the number of MRSA decreased by 1.6 Log (97.3%), which was higher than the antibacterial rate of FMB or H_2_O_2_ alone. This enhancement can be attributed to the Fe_3_O_4_ NPs producing •OH under the acidic biofilm microenvironment. In the FMB + H_2_O_2_ + US + MF group, the number of colonies in the biofilm was reduced by 5.0 Log (99.999%), significantly higher than that of the other groups. This indicates that after the FMB are bound to the biofilm surface by the MF and US is applied, the microjets and shock waves generated by the microbubbles can act on the biofilm at close proximity. Simultaneously, the Fe_3_O_4_ NPs released from the ruptured FMB can penetrate the biofilm under the influence of mechanical forces and generate •OH, which realizes the combination of mechanical disruption and catalytic killing. These results suggest that FMB can efficiently remove MRSA biofilms under MF and US.

### 3.5. Destruction of MRSA Biofilms by FMB in Catheter In Vitro

Medical catheter infections are closely associated with bacterial biofilm formation. In this study, we used silicone catheters with MRSA biofilms to further evaluate the biofilm removal ability of FMB. As shown in Figure S2a, the MRSA biofilm in the Control group showed a distinct purple color after crystal violet staining. In contrast, the MRSA biofilm in the FMB + H_2_O_2_ + US + MF group appeared a light purple, indicating that FMB can effectively disrupt the biofilm structure. The relative biofilm biomass in the FMB + H_2_O_2_ + US + MF group was 71.2%, significantly lower than that of the Control group (Figure S2b). The number of viable bacteria in biofilms was reduced by 5.6 Log (99.999%) in the FMB + H_2_O_2_ + MF + US group (Figure S2c,d), much better than that in the Control group. These results demonstrate that FMB have excellent biofilm removal capabilities under the combined action of US and MF.

### 3.6. Treatment of Catheter MRSA Biofilms Infection by FMB in Mice

**Figure 9:**
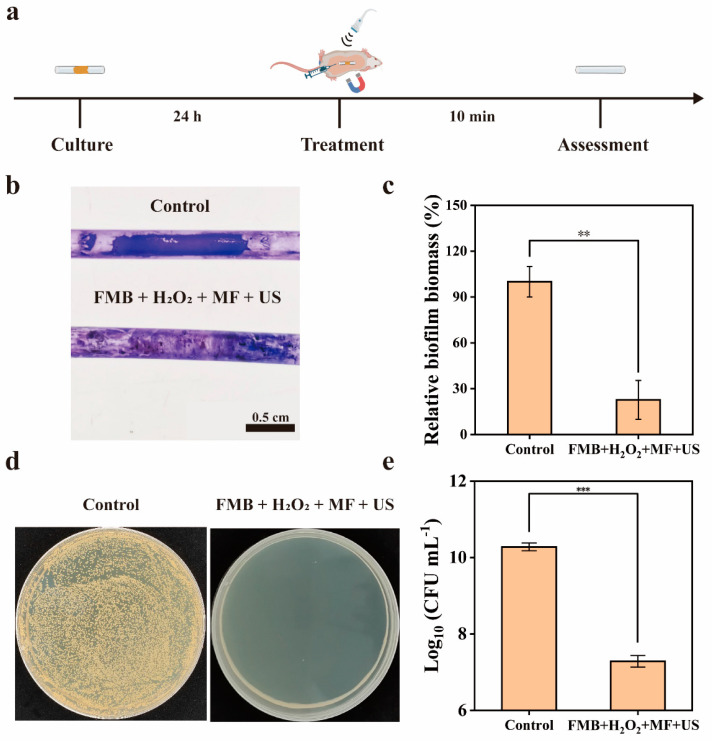
In vivo catheter MRSA biofilm clearance by FMB. (**a**) Schematic of the treatment of catheter biofilms in mice. (**b**) Photographs of crystal violet-stained and (**c**) relative biofilm biomass of catheters after different treatments. (**d**) MRSA colonies on agar plates and (**e**) number of viable bacteria within MRSA biofilm. ** *p* < 0.01, and *** *p* < 0.001.

As shown in Figure 9a, a mouse catheter biofilm infection model was constructed to investigate the antibiofilm effect of FMB under the combined action of US and MF. The MRSA biofilm in the Control group showed a distinct purple color after crystal violet staining, while the catheter in the FMB + H_2_O_2_ + MF + US group appeared significantly lighter in color. This indicates that FMB can effectively disrupt the biofilm under the MF/US. The relative biofilm biomass in the catheter was reduced by 42.7% (Figure 9b,c). The number of viable bacteria within the MRSA biofilm in the FMB + H_2_O_2_ + MF + US group was reduced by about 3.0 Log (99.9%) (Figure 9d,e). These results indicate that FMB can effectively target and remove the catheter-associated MRSA biofilm in mice under the combined effects of MF and US.

### 3.7. Biosafety of FMB

Biocompatibility of materials is an important prerequisite for determining whether they can be used in biomedical applications. First, the cytotoxicity of FMB to human umbilical vein endothelial cells (HUVEC) was investigated by using CCK-8 assay. As shown in Figure S3a, after incubating FMB with HUVEC for 24 h, the cell viability of HUVEC remained above 90% when the concentration of Fe_3_O_4_ NPs in FMB was up to 2 mg/mL, indicating low cytotoxicity of FMB. Next, murine red blood cells (RBCs) were incubated with FMB for 3 h to assess their hemolytic activity. As shown in Figure S3b, the hemolysis rate of RBCs was less than 5% even when the concentration of Fe_3_O_4_ NPs in FMB was as high as 2 mg/mL, suggesting that FMB have a low hemolysis effect.

## 4. Conclusions

In this work, we prepared Fe_3_O_4_ microbubbles (FMB) with mechanical and catalytic properties for the efficient removal of MRSA biofilms using MF and US. FMB have superparamagnetic properties and can be targeted to the biofilm sites in catheters under the guidance of MF. FMB has a nanoparticle shell–air core structure with excellent US-responsive properties, which can be used to destroy the biofilm structure through the cavitation effect. Simultaneously, the released Fe_3_O_4_ NPs provide numerous catalytic sites to generate ROS from H_2_O_2_ and kill the bacteria within the biofilm. In vitro experimental results show that FMB can remove 71.2% of catheter MRSA biofilms using MF/US with H_2_O_2_, and reduce the number of viable bacteria in biofilms by 5.6 Log (99.999%). This work develops a magnetic field-targeted mechanical/catalytic dual-mode removal strategy for bacterial biofilms, which provides a promising solution for effectively addressing bacterial biofilm infection issues.

## Data Availability

The original contributions presented in the study are included in the article/Supplementary Materials, further inquiries can be directed to the corresponding author.

## Supplementary Materials

The following supporting information can be downloaded at: https://www.mdpi.com/article/10.3390/nano14221830/s1. Additional methods: Characterization, Cytotoxicity of FMB, Hemolysis of FMB, and Culture of MRSA biofilms; Additional Figures: Figure S1. MRSA biofilm disruption by Fe_3_O_4_ NPs under MF and US. Figure S2. In vitro catheter MRSA biofilms removal by FMB; Figure S3. Biosafety of FMB.
