# Supplementary material for: Magnetic Field/Ultrasound-Responsive Fe3O4 Microbubbles for Targeted Mechanical/Catalytic Removal of Bacterial Biofilms
Source: Nanomaterials (Basel). 2024 Nov 15;14(22):1830. doi: 10.3390/nano14221830 (PMC11597660; doi:10.3390/nano14221830)
Supplement: Supplementary file 1 [file nanomaterials-14-01830-s001.zip › nanomaterials-3269230-supplementary.pdf]

Supplementary Information

Magnetic field/ultrasound-responsive Fe_3O_4 microbubbles for targeted mechanical/catalytic removal of bacterial biofilms

Liang Lu [1,†], Yuan Liu [1,†], Xiaolong Chen [1], Fengjiao Xu [1], Qi Zhang [1], Zhaowei Yin [2,*] and Lihui Yuwen [1,*]

[1] State Key Laboratory of Organic Electronics and Information Displays, Jiangsu Key Laboratory of Smart Biomaterials and Theranostic Technology, Institute of Advanced Materials (IAM), Nanjing University of Posts and Telecommunications, Nanjing 210023, China

[2] Department of Orthopaedic, Nanjing First Hospital, Nanjing Medical University, Nanjing 210006, China

[*] Correspondence: yzw_1987@126.com (Z.Y.); iamlhyuwen@njupt.edu.cn (L.Y.)

[†] These authors contributed equally to this work

Supplementary Methods:

Characterization

The morphology of the nanomaterials was characterized using a scanning electron microscope (SEM, S-4800, Hitachi, Japan). The ultraviolet-visible-near-infrared absorption spectrometer (UV-vis-NIR, UV-3600, Shimadzu, Japan), vibrating sample magnetometer (VSM, 7404/8604, USA), a multifunctional plate reader (PowerWave XS2, BioTek, USA), an ultrasound therapy instrument (Chattanooga, USA), and a fluorescence microscope (IX71, Olympus, Japan) were used for characterizing the properties of the materials. Microbubbles were prepared using a homogenizer (D-160, DLAB, China),

Cytotoxicity of FMB

Human umbilical vein endothelial cells (HUVEC) were inoculated into 96-well plates at 10^4 per well and incubated at 37°C for 24 h. The upper layer of the medium was removed, and the DMEM medium (serum-free) with different concentrations of FMB was added. The group without FMB was set as the negative control, which was incubated for 24 h. CCK-8 reagent (10 μL) was added to each well, and its absorbance at 450 nm was measured after incubation at 37°C for 3 h. The cell viability was calculated according to the following formula: cell viability = $(A_{sample} - A_{blank}) / (A_{negative} - A_{blank}) \times 100\%$.

Hemolysis of FMB

FMB dispersions at different concentrations (Fe_3O_4 NPs: 0, 0.05, 0.1, 1.0, and 2.0 mg/mL) were mixed with mouse red blood cells (RBCs) saline dispersions and shaken at 37°C. Meanwhile, Triton solution (0.2%) and saline were used as positive and negative controls, respectively. Then, the supernatant was collected by centrifugation and its absorbance at 540 nm was determined. The hemolysis rate was calculated according to the following formula: Hemolysis rate (%) = $(A_{sample} - A_{negative}) / (A_{positive} - A_{negative}) \times 100\%$.

Culture of MRSA biofilms

The methicillin-resistant Staphylococcus aureus strain (MRSA, ATCC43300) was incubated with LB medium at 37° for 12 h to obtain MRSA suspensions.

Culture of MRSA biofilms in a 96-well plate. MRSA suspension (200 μL) with a concentration of 10^7 CFU/mL was added to each well, and then the 96-well plate was placed in an incubator at 37°C for 48 h.

Culture of MRSA biofilms in the confocal dish. MRSA suspension (2 mL) with a concentration of 10^7 CFU/mL was added to a confocal dish and then incubated at 37°C for 48 h.

Supplementary Figures:

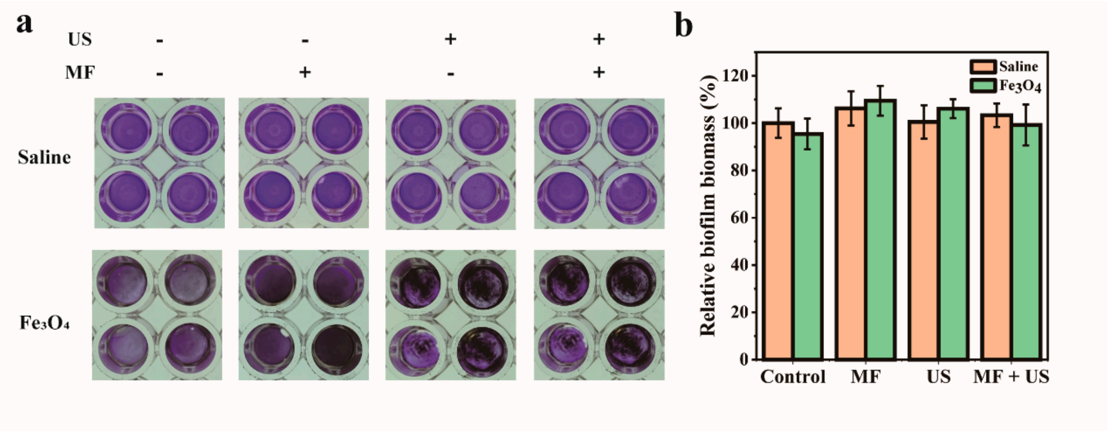

Figure S1 MRSA biofilm disruption by Fe_3O_4 NPs under MF and US. (a) Optical photographs and (b) relative biofilm biomass of MRSA biofilms after crystal violet staining with different treatments.

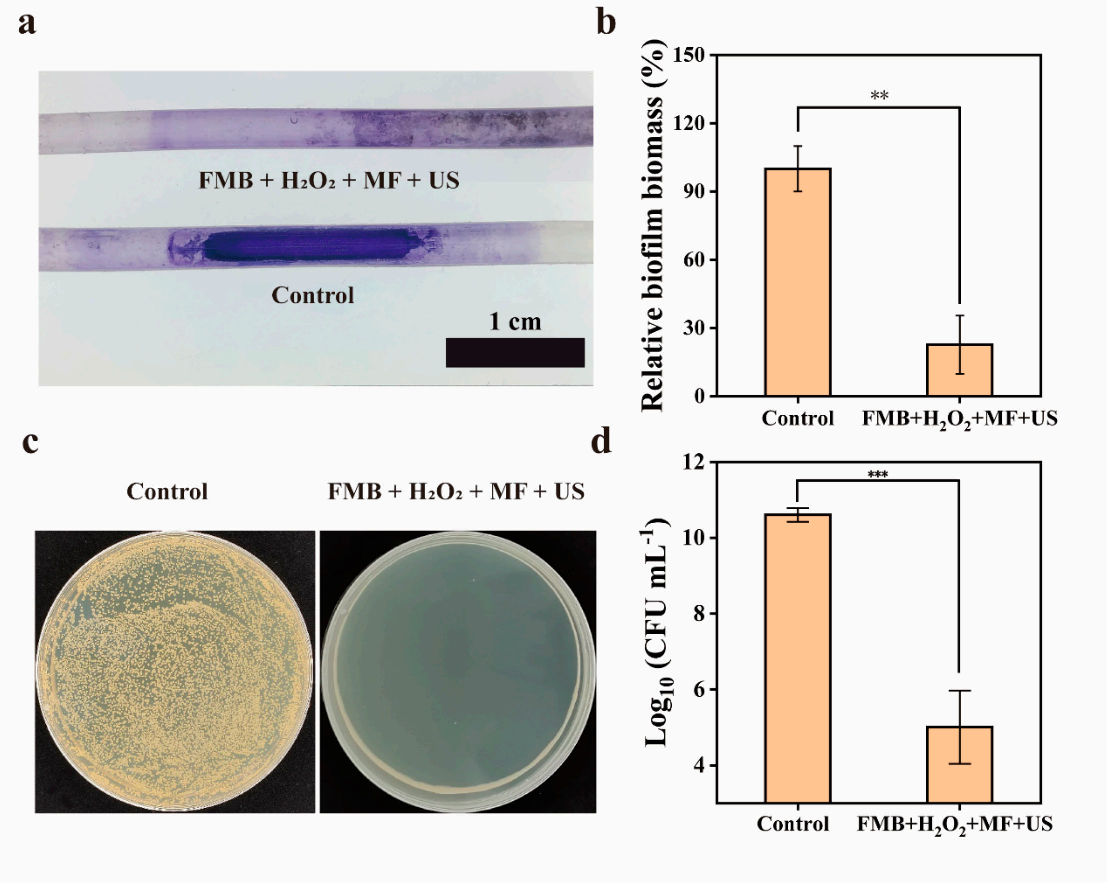

Figure S2 In vitro clearance of catheter MRSA biofilm by FMB. (a) Photographs of catheter biofilms stained by crystal violet. (b) Relative biofilm biomass after different treatments. (c) Photographs of MRSA colonies on agar plates. (d) Number of viable bacteria in MRSA biofilms after different treatments. ** $p < 0.01$, *** $p < 0.001$.

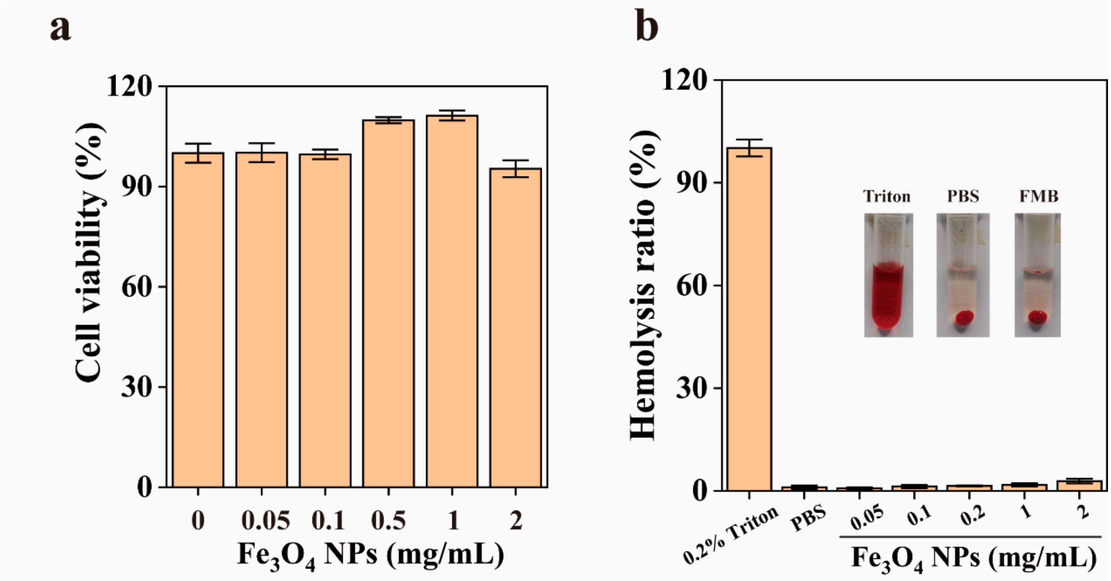

Figure S3 Biosafety of FMB. (a) Cell viability of HUVEC cells after incubation with FMB at different concentrations. (b) Hemolysis rate of RBCs with FMB at different concentrations.
